# Report of the Collective Investigation Committee on Anæsthesia at the Recent Surgical Congress in Berlin

**Published:** 1895-08

**Authors:** 


### Report of the Collective Investigation Committee on Anæsthesia at the Recent Surgical Congress in Berlin.

Professor Gurlt, Berlin, in presenting the report of the committee on anæsthesia, said that, on the proposition of the Society, he had carried out the investigation to a fourth year. In 1893, sixty-three reports had been received, of which nine were from abroad. Fifteen reports had been sent from twenty-one German university clinics. The total of last year's cases was fifty-one thousand eight hundred and forty-six. Of these, thirty-two thousand seven hundred and twenty-three were chloroform administrations, eleven thousand six hundred and seventeen of ether, three thousand eight hundred and ninety-six with ether and chloroform, seven hundred and fifty with chloroform, ether, and alcohol (Billroth's mixture), two thousand seven hundred and sixty-nine with bromide of ethyl, ninety-one with nitrous oxide. The total fatilities were twenty, and of these, seventeen were due to chloroform. The average death-rate was one in two thousand five hundred and eighty-seven administrations. The death-rate from chloroform was one in one thousand nine hundred and twenty-four. When the results of the previous year were added, the totals were one hundred and sixty-three thousand four hundred and ninety three administrations, with sixty-one deaths. The rate of mortality was: chloroform one in two thousand six hundren and fifty-five: chloroform and ether, one in eight thousand and fourteen; Billroth's mixture, one in twenty-six thousand two hundred and sixty-eight. In fact only one death had occurred fron ether narcosis, and that was a case of heart-disease. In correspondence with the low death-rate from ether, its employment had largely increased of late years, from six thousand two hundred in 1892 to eleven thousand six hundred in 1893. Pitchet's purified ice chloroform had been used three thousand eight hundred and ninety times, with two deaths, so it would appear that the dangers of chloroform inhilation did not lie in any accidental impurity; indeed the facts seem to point the other way, and that the danger was directly proportionate to the purity of the chloroform. This consideration had lately led to a different mode of administration. It had been given more slowly, and occasionally atropine and cocaine had been used with it. As regarded accidents, two hundred and fifty-five severe cases of asphyxia had occurred, and tracheotomy had to be performed three times. Konig's cardiac message, so called was frequently, used, but not always with success. Ether had, therefore, shown itself the least dangerous anæsthetic, ten times less dangerous than chloroform, but it was not without its shady side: it was dangerous in lung-affections. It was agreed to still continue the investigation.

